# Ultrafast X-ray imaging of laser–metal additive manufacturing processes

**DOI:** 10.1107/S1600577518009554

**Published:** 2018-08-14

**Authors:** Niranjan D. Parab, Cang Zhao, Ross Cunningham, Luis I. Escano, Kamel Fezzaa, Wes Everhart, Anthony D. Rollett, Lianyi Chen, Tao Sun

**Affiliations:** a X-ray Science Division, Advanced Photon Source, Argonne National Laboratory, Argonne, IL 60439, USA; b Department of Materials Science and Engineering, Carnegie Mellon University, Pittsburgh, PA 15213, USA; c Department of Mechanical and Aerospace Engineering, Missouri University of Science and Technology, Rolla, MO 65409, USA; d Department of Energy’s Kansas City National Security Campus, Managed by Honeywell Federal Manufacturing and Technologies, Kansas City, MO 64147, USA

**Keywords:** X-ray imaging, laser powder-bed fusion, particle ejection, melt pools, vapor depressions, additive manufacturing

## Abstract

The high-speed synchrotron X-ray imaging technique was synchronized with a custom-built laser-melting setup to capture the dynamics of laser powder-bed fusion processes *in situ*. Various significant phenomena, including vapor-depression and melt-pool dynamics and powder-spatter ejection, were captured with high spatial and temporal resolution. Imaging frame rates of up to 10 MHz were used to capture the rapid changes in these highly dynamic phenomena. At the same time, relatively slow frame rates were employed to capture large-scale changes during the process. This experimental platform will be vital in the further understanding of laser additive manufacturing processes and will be particularly helpful in guiding efforts to reduce or eliminate microstructural defects in additively manufactured parts.

## Abstract 2

The high-speed synchrotron X-ray imaging technique was synchronized with a custom-built laser-melting setup to capture the dynamics of laser powder-bed fusion processes *in situ*. Various significant phenomena, including vapor-depression and melt-pool dynamics and powder-spatter ejection, were captured with high spatial and temporal resolution.

## 1. Introduction   

Powder-bed additive manufacturing (AM) processes selectively melt or bind particles in successive thin layers of powder materials to build three-dimensional parts. They offer various advantages over conventional manufacturing methods, such as manufacturing complex parts directly from a design without the requirement for tooling, and on-demand manufacturing. This reduces the inventory of spares and decreases the lead time (DebRoy *et al.*, 2018 ▸). As a result of these advantages, AM of metallic materials is growing rapidly in the medical, aerospace, automobile and defense industries (Wohlers & Caffrey, 2015 ▸; Bourell, 2016 ▸).

Currently, laser powder-bed fusion (LPBF) is the most popular method for manufacturing metal parts (Rosen, 2007 ▸; Campbell *et al.*, 2011 ▸). In a typical LPBF process, a laser beam is scanned across a layer of powder with a thickness of around 50 µm, which is laid on top of a substrate. The laser beam selectively melts the powder particles and the top of the substrate, with a typical melt-pool width of around 200 µm. The subsequent cooling of the molten material results in a new layer of solid metal (Santos *et al.*, 2006 ▸; Kruth *et al.*, 2007 ▸). The extremely high heating and cooling rates cause many dynamic and transient phenomena in LPBF processes, including melting and partial vaporization of powders, flow of molten metal, powder ejection and redistribution, fast solidification and non-equilibrium phase transitions (Das, 2003 ▸; King *et al.*, 2014 ▸; Khairallah *et al.*, 2016 ▸; Matthews *et al.*, 2016 ▸). Laser light impinging on the powder bed and substrate leads to local melting of the material, subsequently forming a melt pool directly underneath and behind the laser. Moreover, the high power density commonly leads to a vapor depression or keyhole, as our results clearly demonstrate, which influences the size and shape of the melt pool. The shape of the melt pool in turn affects the size and shape of the resultant grains (DebRoy *et al.*, 2018 ▸), which typically arise epitaxially from the heat-affected zone and grow parallel to the temperature gradient. The motion of the melt pool also affects the solidification rate (DebRoy *et al.*, 2018 ▸).

Thanks to the extremely high cooling and solidification rates, three-dimensional-printed components also exhibit various non-equilibrium phases (DebRoy *et al.*, 2018 ▸). If the melt-pool surface is heated above the boiling point, the recoil momentum produced by the vaporized metal exerts a force on the molten material, and a cavity or vapor depression forms underneath the laser spot (King *et al.*, 2014 ▸). The formation of a laser cavity, or keyhole, further enhances laser absorption because the light drills deeper into the material (King *et al.*, 2014 ▸) and the heat source becomes, effectively, a moving line source as opposed to the commonly assumed point source. Unstable collapse of the cavity can leave voids and defects in the laser path (King *et al.*, 2014 ▸), which can have negative effects on mechanical properties such as fatigue life (Fadida *et al.*, 2015 ▸). Occasionally, molten metal is entrapped and ejected by the metal vapor, which leads to spatter. Spatter particles eventually fall back onto the powder bed and may subsequently contribute to structural defects (Slotwinski *et al.*, 2014 ▸; Nandwana *et al.*, 2016 ▸; Ly *et al.*, 2017 ▸).

For LPBF processes, the dynamics of the powder particles also plays an important role in determining the quality of the final product. Intact powder particles are trapped by the metal vapor which is ejected upwards from the powder bed and backwards from the laser scanning direction (Matthews *et al.*, 2016 ▸). If a sufficiently large fraction of powder particles are ejected from the powder bed, the building process may be impacted negatively (Matthews *et al.*, 2016 ▸; Slotwinski *et al.*, 2014 ▸; Nandwana *et al.*, 2016 ▸). Further, if the ejected particles fall back onto the active area of the powder bed, they affect the spreading of the powder for the next layer and thus contribute to structural defects (Slotwinski *et al.*, 2014 ▸). Even if the particles and spatter fall away from the active building area, they adversely affect powder recycling by generating agglomerates (Slotwinski *et al.*, 2014 ▸; Nandwana *et al.*, 2016 ▸), which in part explains why the LPBF machine manufacturers pay attention to gas flow in the chamber. Further, particles close to the melt track are consumed through direct contact with liquid metal (Matthews *et al.*, 2016 ▸). The melting and ejection of particles adjacent to the melt track lead to the formation of a denudation zone (Matthews *et al.*, 2016 ▸), and such zones lead to the formation of elongated pores (Thijs *et al.*, 2010 ▸) and track asymmetry (Matthews *et al.*, 2016 ▸).

It is clear that the aforementioned physical phenomena ultimately affect the quality and properties of parts manufactured using powder-bed fusion processes (Cunningham *et al.*, 2016 ▸, 2017 ▸; Li *et al.*, 2016 ▸; Collins *et al.*, 2016 ▸). However, it is extremely challenging to monitor these phenomena experimentally because of the highly localized and extremely fast interaction of the laser beam with the metal powders. Various imaging methods have previously been used to study LPBF processes *in situ* (Everton *et al.*, 2016 ▸). The majority of these studies have used high-speed visible-light (Matthews *et al.*, 2016 ▸; Ly *et al.*, 2017 ▸; Scipioni Bertoli *et al.*, 2017 ▸; Trapp *et al.*, 2017 ▸; Bidare *et al.*, 2017 ▸, 2018 ▸) or thermal imaging (Pavlov *et al.*, 2010 ▸; Furumoto *et al.*, 2013 ▸; Lane *et al.*, 2016 ▸; Fox *et al.*, 2017 ▸). High-speed visible-light imaging was used to study particle entrainment and denudation (Matthews *et al.*, 2016 ▸), spatter formation (Ly *et al.*, 2017 ▸), and laser–melt-pool interactions (Scipioni Bertoli *et al.*, 2017 ▸). High-speed visible-light Schlieren imaging was also used to study the metal-vapor jetting generated due to evaporation of the material underneath the laser beam (Bidare *et al.*, 2017 ▸, 2018 ▸). Two-color pyrometry (Pavlov *et al.*, 2010 ▸; Furumoto *et al.*, 2013 ▸), in-line thermal imaging (Fox *et al.*, 2017 ▸) and off-axis thermal imaging (Lane *et al.*, 2016 ▸) have been used to monitor the melt-pool temperature during the build process. The melt-pool geometry has also been studied using in-line coherent imaging (Kanko *et al.*, 2016 ▸). The main advantage of using high-speed visible-light or thermal imaging is the potential for integrating these techniques with the AM machines for process control during the build, yet both visible-light and thermal imaging are limited to surface monitoring and cannot be used to quantify subsurface features such as the vapor depression and melt pool, particularly their morphologies along the build direction. Visible-light monitoring of the ejected powder and spatter particles is also challenging because of the uneven illumination of the particles that depends on the temperature and depth of focus of the imaging system.

To overcome these issues, a high-speed X-ray imaging system was developed to monitor the LPBF process (Zhao *et al.*, 2017 ▸). In our previous work, the laser spot was stationary with respect to the specimen and hence only the ‘spot-welding’ mode of the laser-melting process was investigated. In this paper, our previous high-speed X-ray imaging system has been upgraded to include a laser scanner to reproduce the actual LPBF process. Recently, two other *in situ* X-ray imaging systems have been developed and used by other teams to study the laser-melting processes in a Ti–6Al–4V alloy (Calta *et al.*, 2018 ▸) and an Invar 36 alloy (Leung *et al.*, 2018 ▸) with relatively slow recording rates. In this contribution, selected experiments were performed using an ultrafast imaging camera with a temporal resolution reaching 100 ps and a recording rate reaching 6.5 MHz. Some extremely fast physical processes involved in LPBF that will require such ultrafast recording speeds were identified. A framework for imaging heavier structurally relevant materials such as nickel superalloys and stainless steel is also proposed. This experimental framework will be vital in improving the fundamental understanding of the physics that governs the LPBF process and will subsequently help in improving the quality of parts manufactured using the LPBF processes.

## 2. Materials and methods   

### 2.1. Materials   

Base plates and miniaturized powder-bed samples were prepared from three different metallic alloys: aluminium alloy (Al–10Si–Mg), titanium alloy (Ti–6Al–4V) and nickel alloy (Inconel 718). These three alloys are in common use for commercial laser-melting powder-bed manufacturing (Frazier, 2014 ▸; Herzog *et al.*, 2016 ▸; Sames *et al.*, 2016 ▸). Miniaturized plate specimens manufactured from aluminium alloy and nickel alloy were used to study the interaction of the laser beam with the substrate. The materials were first procured commercially as larger plates and appropriate specimens were machined from these plates. The plate specimens were 2.9 mm wide and 50 mm long, and 800 µm and 380 µm thick for the aluminium and nickel alloys, respectively. The specimens were oriented in the vertical position.

**Figure 1:**
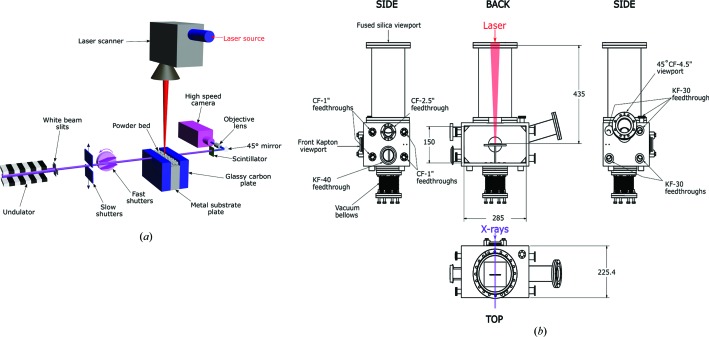
(*a*) A schematic of the laser AM experiments synchronized with the high-speed X-ray imaging setup. A short-period undulator was used to generate a pseudo-pink beam with a first harmonic energy of 24.4 keV. Two sets of shutters were used to control the X-ray exposure time window. The laser impinged on the specimen from the top. The X-ray beam penetrated the sample from the side and was subsequently converted to visible radiation by the scintillator. Visible-light images were directed to the high-speed camera through a mirror and objective lens. (*b*) A schematic of the experimental chamber. The laser and X-ray paths are not to scale.

Miniature aluminium and titanium alloy powder-bed systems were used to mimic the laser-melting processes observed in commercial AM machines. They consisted of a metal base (800 µm thick for aluminium alloy and 450 µm thick for titanium alloy; 2.9 mm wide and 50 mm long) sandwiched between two glassy carbon plates (1 mm thick, 3.0 mm wide and 50 mm long; Grade 22, Structure Probe Inc., USA). A uniform layer of powder (∼100 µm thick) was spread manually on top of the metal base. A schematic of the powder-bed specimen is presented in Fig. 1 ▸(*a*). Details of the metal substrates and powders, including vendors and particle sizes for each material, are presented in Table 1 ▸. Both plate and powder-bed samples were maintained at room temperature prior to the experiment.

### 2.2. Laser setup   

A custom-built experimental laser platform was developed to perform the high-speed X-ray experiments. This setup was upgraded from a previously reported laser platform (Zhao *et al.*, 2017 ▸). Figs. 1 ▸(*a*) and 2 ▸ show a schematic of the experimental setup and photographs of the laser setup, respectively. The laser system consists of an ytterbium fiber laser source (IPG YLR-500-AC, IPG Photonics, Oxford, Massachusetts, USA) integrated with a laser scanner (Intelli*SCAN*_de_ 30, SCANLAB GmbH, Puchheim, Germany). The fiber laser provided pure Gaussian beam profiles and was operated in single mode. The wavelength and maximum power of the laser were 1070 nm and 520 W, respectively. At the focal point, the beam spot size was approximately 50 µm. In the current experiments, larger spot sizes (*e.g.* 100 µm) were achieved by defocusing the laser beam below the laser-beam focal plane. The laser can be operated in both continuous wave (CW) and modulation modes with frequencies up to 50 kHz. All experiments in this study were performed in CW mode. The laser source was connected to the laser scanner through a feeding fiber and a collimator. The laser scanner manipulates the laser beam using a system of rotating mirrors driven by galvanometers. The specified maximum scan speed was 0.7 m s^−1^, although higher scan speeds are feasible. The parameters for the laser and scanner (laser power, scan speed, scan length and delay times) were controlled through a desktop computer using proprietary software (*laserDESK*, SCANLAB GmbH). During the experiments, the laser was operated in the ‘line-scan’ mode, where the laser was translated in a straight line along the top of the plate or the powder bed at specified power and velocity values. The scan lengths and locations were selected such that the parts of the scan that were to be observed (start, steady-state motion or end) were in the X-ray window. The scanner was also fitted with an in-line CCD camera (UI-5240CP-M-GL, iDS Imaging Development Systems GmbH, Obersulm, Germany), which was initially aligned with the laser and subsequently used to align the specimens with the laser beam.

The specimens were placed inside a stainless steel vacuum chamber (inner dimensions: length 285 mm, height 150 mm, width along the X-ray propagation direction 200 mm, wall thickness 12.7 mm) for the experiments. A schematic of the experimental chamber is presented in Fig. 1 ▸(*b*). A fused-silica window (diameter 152.4 mm) was located on the top of the chamber for passage of the laser beam. The laser scanner was located on the top of the fused-silica window, which was separated from the top of the chamber box by a vacuum flange 350 mm long. The length of the flange was selected such that the distance between the scanner and the sample was approximately equal to the working distance of the scanner *f*–θ lens. Two additional viewports were incorporated in the front and back (front diameter 63.5 mm, back diameter 152.4 mm) of the chamber and sealed using 127 µm-thick Kapton film. Since this chamber will also be used to perform high-speed diffraction studies in future, the size of the back window was chosen such that diffracted photons could also be captured. The chamber was connected to a mechanical vacuum pump (model XDS10, Edwards Vacuum, Sanborn, New York, USA) and a fill line from an argon cylinder (maximum pressure 13.8 MPa) through a KF-40 vacuum flange for pumping and purging the chamber. Additionally, a pressure transducer (model KJL275808LL, Kurt Lesker, Jefferson Hills, Pennsylvania, USA) was attached to the same connector to gauge the pressure inside the chamber. In the current configuration, the vacuum pump was capable of pumping the chamber to low vacuum (∼13.33 Pa). After pumping out, the chamber was back-filled with argon to atmospheric pressure to prevent potential oxidation of the metals.

The chamber was also equipped with additional feedthroughs on the left- and right-hand sides (with respect to the X-ray propagation direction) for electronic control and feedback (right: four CF-1", one CF-2.75" and one KF-40 flange; left: four KF-30 flanges). One of the CF-1" feedthroughs was used to control two red LEDs, which were used for illumination during alignment of the sample using the in-line CCD camera. Additionally, an inclined viewport (diameter 114.3 mm, inclination angle 45°) was integrated on the left-hand side for the potential observation of the top surface of the sample. On the bottom of the chamber, 177.8 mm-long and 69.8 mm inner-diameter vacuum bellows were connected to the chamber on the one side and to a three-axis translational stage assembly on the other. The translational stage assembly was composed of three one-axis translational stages equipped with stepper motors (model XA07A-R102, Kohzu Precision, Kawasaki Kanagawa, Japan). The ranges of motion for the in-plane translation and vertical stages were 20 and 10 mm, respectively. A vertical post was fixed on the stage assembly and fed through the bellows into the chamber. An aluminium breadboard was fixed on the other side of the vertical post to position the specimens. The horizontal translational stage assembly was used to align the specimens with the laser spot prior to the experiments. The vertical stage was used to control the distance between the scanner and the specimen, which controlled the laser spot size. The chamber and the translation stages were placed on top of heavy duty vertical and horizontal stages, which were used to align the laser with the X-ray beam.

### 2.3. High-speed synchrotron X-ray imaging setup   

**Figure 3:**
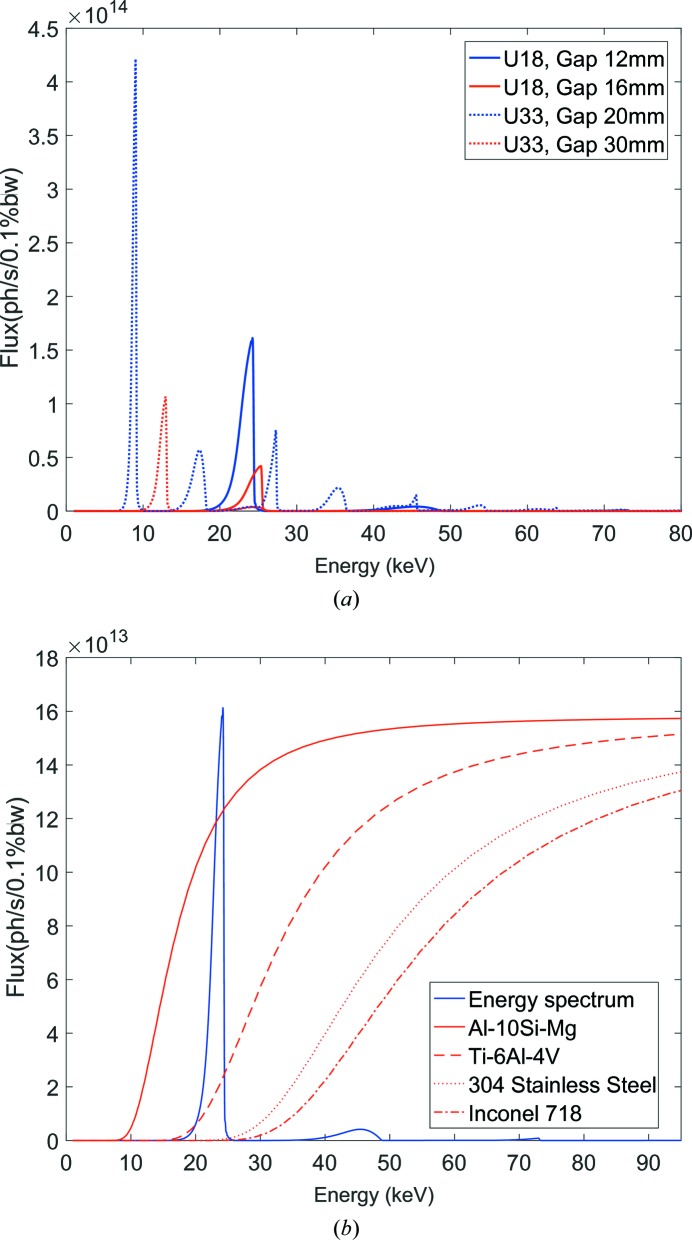
(*a*) Energy spectra of the X-ray beams generated with different undulator conditions. (*b*) X-ray transmission through different metallic materials with 500 µm thickness. Also shown here is the energy spectrum of the X-ray beam generated using the 1.8 cm period undulator with the gap set to 12 mm.

The high-speed synchrotron X-ray full-field imaging experiments were performed on beamline 32-ID-B at the Advanced Photon Source (APS), Argonne National Laboratory. A schematic and photograph of the high-speed imaging setup are presented in Figs. 1 ▸(*a*) and 2 ▸(*a*), respectively. A short-period (18 mm) undulator with the gaps set between 12 and 16 mm was used for the current experiments. Beamline 32-ID is also equipped with a long-period undulator (33 mm) which may be used for different experimental conditions in future. The energy spectra for two typical gaps for both undulators are presented in Fig. 3 ▸(*a*) and the transmission spectra through relevant metallic materials are presented in Fig. 3 ▸(*b*). The transmitted intensity dropped significantly for all energies as the density of the material was increased. This reduced transmission affected the signal-to-noise ratio for the high-speed images and hence required longer exposure times for experiments with heavier materials such as nickel alloys and stainless steel. A set of horizontal and vertical white-beam slits was used to collimate the X-ray beam and control its size. For the current experiments, the slit dimensions were typically set to 1.5 mm × 1.5 mm. The integrated flux values for the specified gaps and slit openings are presented in Table 2 ▸. Since the photon flux was concentrated at the first-harmonic energy (less than 4% of the overall flux was at higher harmonics) for the short-period undulator, the incident X-ray beam behaved similarly to a pink beam with an energy bandwidth of ∼7%. The distance between the X-ray source and the specimen was around 38 m. The distance between the specimen and the detector (scintillator) was around 400 mm.

**Figure 4:**
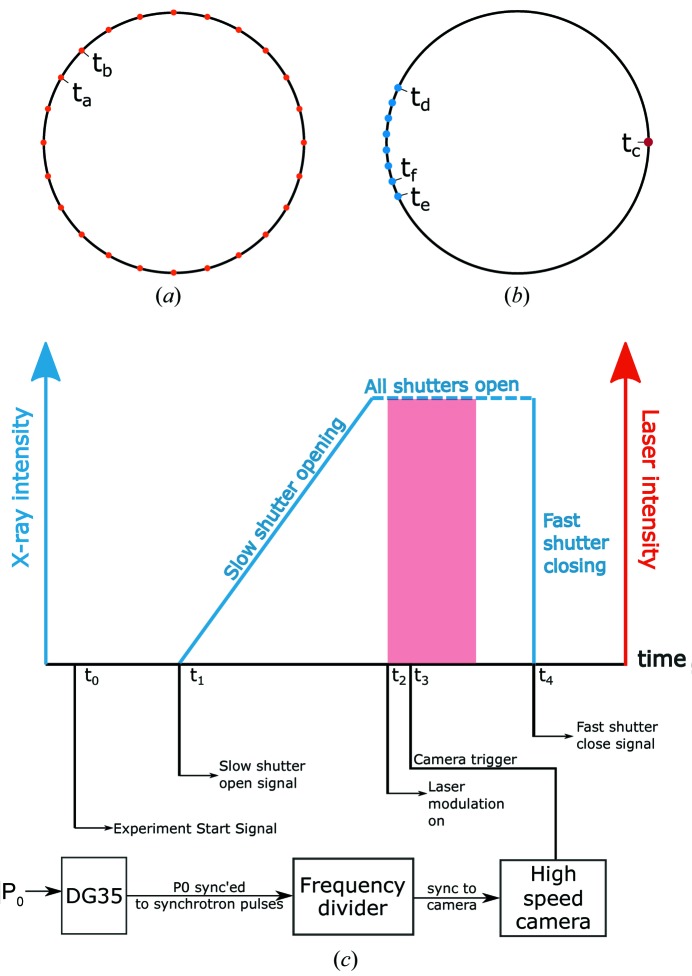
(*a*) A schematic of the electron fill pattern in APS standard mode (24-bunch mode). The bunch separation was 153 ns (t_a_–t_b_). The length of each pulse was 33.5 ps. (*b*) The electron fill pattern for APS hybrid mode. The separation between the singlet and the superbunch (t_c_–t_d_ and t_e_–t_c_) was 1.58 µs. The separation between septuplet groups (t_e_–t_f_) was 51 ns. The total duration for the septuplet bunch (t_d_–t_e_) was 500 ns. The lengths of the singlet and septuplet bunches were 50 and 27 ps, respectively. (*c*) Timing and synchronization schemes for the laser AM experiments performed in hybrid mode. P_0_ are the synchrotron radio-frequency pulses (master clock), separated by 3.68 µs. For experiments performed in standard mode, synchronization with the master clock was not required.

The temporal structure of X-ray pulses corresponds to the time structure of the electron bunches, which depends on the operation mode of the APS. For dynamic measurements, the electron bunch current, pulse width and pulse separation, along with the synchronization of the X-ray pulses with the laser experiments and detectors, must be carefully considered. The electron bunches (pulse train) are maintained in a circular storage ring with a circumference of 1104 m. The time required for the electrons to complete one revolution around the storage ring is 3.683 µs. The number of photons in an X-ray pulse scales linearly with the bunch current. Two different storage-ring operation modes were used in the current experiments. The standard 24-bunch mode was used for ultrafast recordings (1.08 and 10 MHz) and the hybrid mode was used for comparatively slower recordings (30 and 50 kHz). Schematics of the bunch structures for the 24-bunch and hybrid modes are presented in Figs. 4 ▸(*a*) and 4 ▸(*b*), respectively. In the 24-bunch mode, the storage ring contained 24 equidistant electron bunches with equal current (approximately 4.25 mA each), equivalent to a total current of approximately 102 mA. The separation between consecutive bunches was 153 ns. The r.m.s. pulse width of the X-rays emitted by each bunch was 33 ps. In hybrid mode, a single bunch containing a 16 mA current (singlet) was isolated from the remaining bunches by a symmetrical 1.594 µs gap. The remaining current was distributed into eight groups (super-bunch) of seven consecutive bunches (septuplet), with a current of 11 mA per group, a periodicity of 68 ns and a gap of 51 ns between groups. The total length of this bunch train was 500 ns. The r.m.s. pulse width of the X-rays for the singlet bunch was 50 ps and for the septuplet group was 27 ps.

The X-rays generated by the undulator pass sequentially through the white-beam slits, slow shutter, fast shutter, specimen and detector. The slow and fast shutters were used to control the X-ray open time-window position with respect to the laser-on and experiment time window and the camera recording time window. The slow shutter comprised two water-cooled copper blocks mounted on fast-response linear actuators with opening and closing times of around 50 ms. The fast shutter was manufactured by gluing two diamond-shaped tungsten blocks onto a goniometer with opening and closing times of around 500 µs.

In the current experiments, the recorded X-ray images contain both absorption and phase contrast. Absorption contrast corresponds to differences in the transmitted intensities as X-rays are attenuated (absorbed) by materials in the sample. X-ray phase contrast is related to the Laplacian of the phase of the wavefront after passing through a sample containing materials with different refractive indices, which provides greater edge contrast, particularly for lighter mater­ials and sharp interfaces (Wilkins *et al.*, 1996 ▸; Murrie *et al.*, 2014 ▸). A single-crystal Lu_3_Al_5_O_12_:Ce scintillator was used to convert the transmitted X-ray signal to light at visible wavelengths. The scintillator had a diameter of 10 mm and thickness of 100 µm. The decay time of the scintillator was around 45–55 ns and its emission spectrum peaked at 530 nm (Luo *et al.*, 2012 ▸; Olbinado *et al.*, 2017 ▸). In previous studies, the signal from the Lu_3_Al_5_O_12_:Ce scintillator was observed to decay to around 40% of its original value in 153 ns (interpulse duration), thus giving a long afterglow effect (Olbinado *et al.*, 2017 ▸). However, in this study, no ghost images were observed from the afterglow effect, even at ultrafast recording speeds. The converted optical photons were relayed to the high-speed camera through a 45° mirror, a 10× microscope objective (numerical aperture 0.28) and a tube lens. Two different high-speed cameras were used to record the images: a Photron FastCam SA-Z (Photron Inc., Tokyo, Japan) for recording speeds between 30 and 50 kHz, and a Shimadzu HPV-X2 (Shimadzu Corp., Kyoto, Japan) for recording speeds of 1.08 to 10 MHz. Table 3 ▸ provides details of the settings for each camera. The Photron-SA-Z camera uses a continuous-readout CMOS image sensor (Olbinado *et al.*, 2017 ▸). The Shimadzu HPV-X2 high-speed camera uses an ultrahigh-speed CMOS image sensor with on-chip analog memories placed on the edges of the imaging pixel array (Tochigi *et al.*, 2013 ▸; Kuroda & Sugawa, 2018 ▸). This camera has a capability of a readout speed of 1 Tpixel s^−1^ in burst mode, thus enabling full-resolution image recording at 10 MHz for 128 frames (Tochigi *et al.*, 2013 ▸). Previously, researchers have used the Shimadzu HPV-X2 at 10 MHz recording rates to study various rapid phenomena including material deformation (Sutton *et al.*, 2018 ▸), plasma deflagration (Underwood *et al.*, 2017 ▸), cavitation bubble luminescence (Supponen *et al.*, 2017 ▸) and bubble-collapse shock waves (Johansen *et al.*, 2017 ▸).

**Figure 5:**
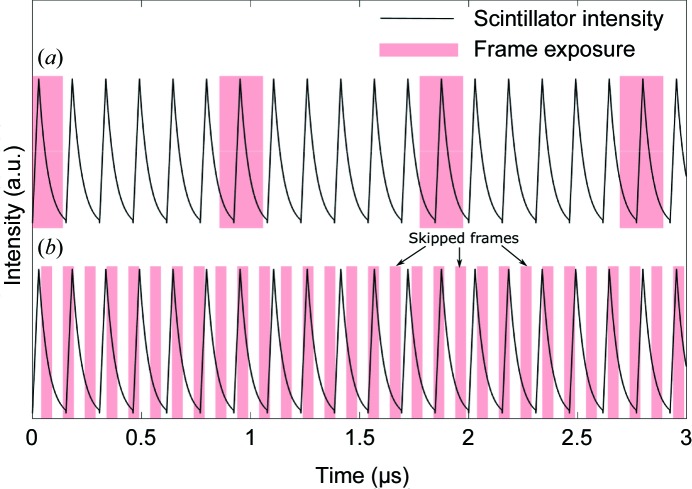
Schematics of the temporal structure of the frame exposure times and the scintillator intensity for (*a*) 1.08 MHz (synchronized) and (*b*) 10 MHz recording (mismatch). The scintillator intensity showed an exponential decay from the maximum intensity with a time constant of 42 ns (Luo *et al.*, 2012 ▸). For 1.08 MHz recording, the frame exposure times were synchronized with the X-ray pulses. For 10 MHz recording, the X-ray pulses were temporally mismatched with the frame exposure times, which resulted in flashing and repeated images for consecutive frames, thus giving the true recording rate of 6.5 MHz.

The temporal resolution values presented in Table 3 ▸ refer to the exposure time or X-ray integration time for a single frame. For the hybrid mode, the images were recorded using either the singlet or the superbunch mode, which translated to a temporal resolution of about 100 ps or an X-ray integration time of about 500 ns. For recording at 1.08 MHz using the 24-bunch mode, the single-pulse X-ray integration time was around 100 ps and the frame exposure time was synchronized with the X-ray pulses, as shown in Fig. 5 ▸(*a*). For the 10 MHz recording rate, the inter-frame separation (100 ns) was shorter than the pulse separation in the 24-bunch mode (153 ns). However, the scintillator decay time was sufficiently long that some illumination was still available for recording the image even when an X-ray pulse was not impinging on the sample and scintillator during the frame exposure time. This mismatch between the X-ray pulses and the frame exposure is presented in Fig. 5 ▸(*b*). Since some frames only captured the afterglow image from the previous X-ray pulse exposure (skipped frames are labeled in Fig. 5 ▸ *b*), the mismatch between the X-ray pulses and the frame exposure resulted in an equivalent frame separation of 6.5 MHz, which is the pulse frequency of the synchrotron source. The mismatch between the X-ray pulses and the frame exposure times also resulted in uneven illumination across consecutive frames during the recording, and hence apparent ‘flashing’ was observed during playback. Since the illumination was still provided by a single pulse in the 24-bunch mode, the temporal resolution for the 10 MHz recording was also around 100 ps.

### 2.4. Experimental procedure   

For a successful experiment, the timing sequence and synchronization of the X-ray open–close time window (X-ray shutters), the system trigger time, the actual laser-melting event and image recording were critical. In both standard and hybrid modes, the actual line scan was delayed by 500 ms from the start signal (*t* = 0) to accommodate the time required to open the slow shutter and also the inherent delays present in the laser-scanner setup. At *t* = 450 ms, a TTL pulse signal was sent using a delay generator (DG35, Stanford Research Systems, Sunnyvale, California, USA) to the slow shutter to initiate the opening sequence, such that the slow shutter was fully open during the line scan. At *t* = 500 ms, when the line scan was initiated, another DG was used to send the trigger to the high-speed camera. For ultrafast recording speeds (24-bunch mode), the DG signal was relayed directly to the camera and the frames were recorded as per predefined recording speeds and exposure times without forced synchronization with the X-ray pulses. For comparatively slower recording speeds (hybrid mode), the frames were synchronized with the X-ray pulses either with the singlet or the superbunch. Finally, the fast shutter was activated through a DG to close after the event was completed. The closing of the fast shutter marked the end of the experiment.

## 3. Results and discussion   

### 3.1. Observation of key phenomena in LPBF processes   

**Figure 6:**
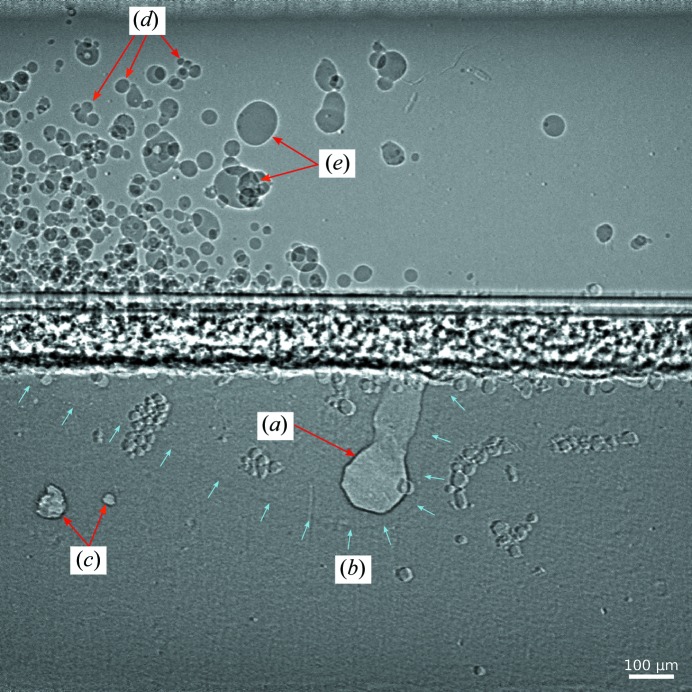
A high-speed X-ray image from a representative LPBF experiment. (*a*) The vapor depression, (*b*) the melt pool, (*c*) keyhole porosity, (*d*) ejected powder and (*e*) spatter or molten metal ejected from the melt pool. The substrate and the powder were composed of Al–Si10–Mg. The substrate thickness (along the X-ray beam direction) was 800 µm and the powder size was between 15 and 45 µm. The laser power was set at 520 W and the scanning speed was 0.6 m s^−1^. X-ray images were recorded at 30173 Hz with an effective exposure time of 100 ps. Some powder clusters adhered to the outside of the holder and did not contribute to the laser-melting and solidification processes. These clusters can be observed on the left and right of the vapor depression.

The *in situ* observation of key physical phenomena (melt-pool dynamics, laser cavity or vapor depression, and powder and spatter dynamics) is vital for studying the underlying physics of LPBF processes and for controlling the defect density in parts manufactured using LPBF. A representative X-ray image from a powder-bed experiment for the Al–10Si–Mg alloy obtained using the Photron FastCam SA-Z camera is presented in Fig. 6 ▸. The aforementioned key phenomena are all clearly evident. Observation of the vapor depression and melt pool was feasible due to the differences in density of the solid, liquid and gaseous phases of the material. X-ray imaging is an ideal technique to discern the subtle density differences between the liquid and the solid state. Further, the porosity generated by the laser melting can also be readily observed.

**Figure 7:**
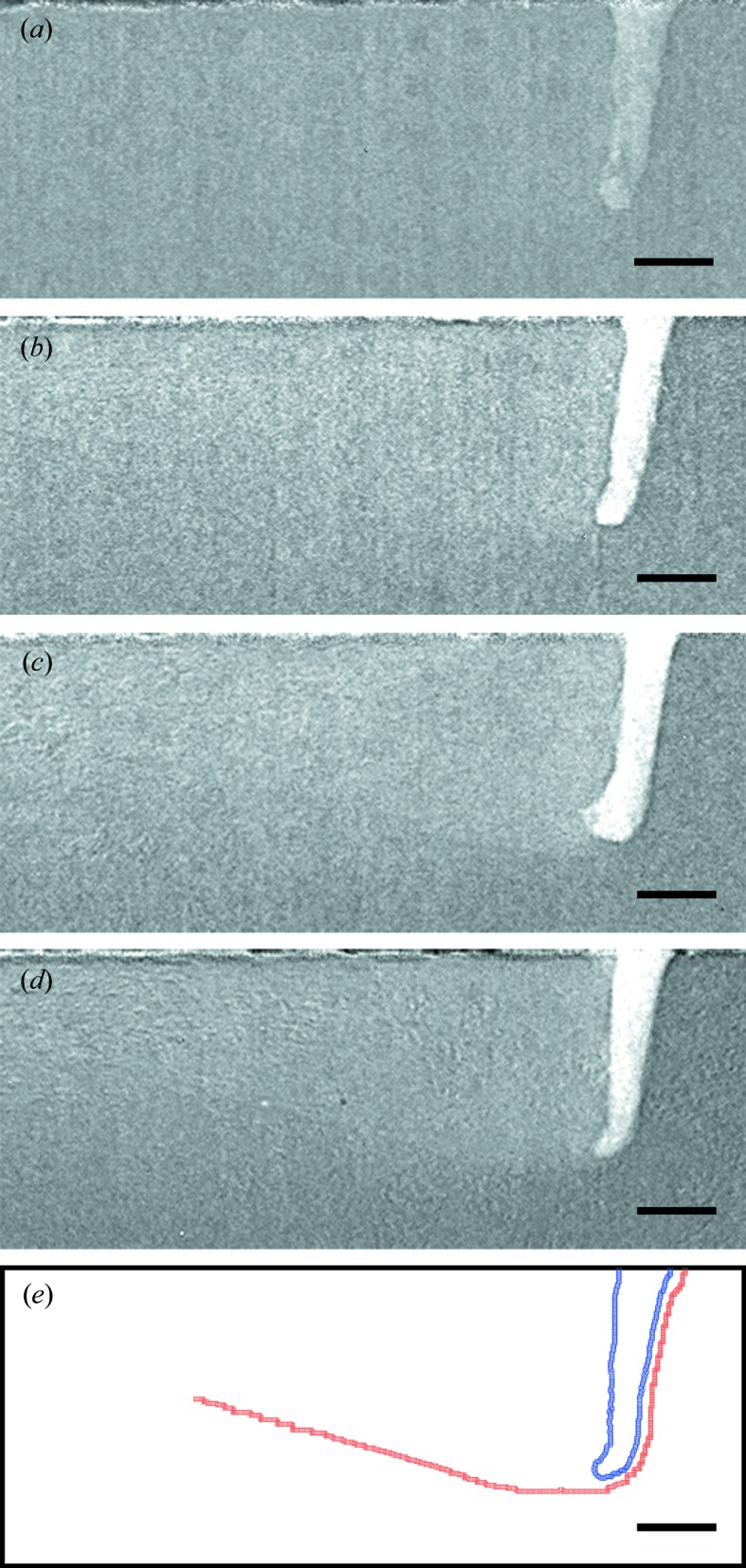
Influence of camera exposure time on imaging of the solid–liquid interface during the laser AM process. (*a*) 1 µs. (*b*) 5 µs. (*c*) 10 µs. (*d*) 20 µs. (*e*) The solid–liquid interface (red squares) and liquid–gas interface (blue circles). The laser was scanned from left to right in this and all experiments. The vapor hole (depression) is comparable in diameter with that of the laser beam at the top surface of the metal. The thickness of the liquid layer in front of the vapor depression is small, whereas the melt pool extends several hundred micrometres behind the depression. The sample thickness (along the X-ray beam direction) is 380 µm. The laser power was 260 W and the scanning speed was 500 mm s^−1^. The scale bars are 100 µm.

The flux of photons transmitted through denser materials such as an Ni-based superalloy (Inconel 718) was much lower than that for lighter materials such as the aluminium alloy (Al–10Si–Mg), as shown in Fig. 3 ▸(*b*). Hence, features with small density differences, such as the contrast between the melt pool and the surrounding solid, cannot be identified at short exposure times. This can be clearly observed in Fig. 7 ▸(*a*), where the vapor depression can be identified because the density difference between the liquid and gas phases is large. However, the melt pool cannot be visualized with sufficient contrast. As the exposure time was increased sequentially from 1 to 20 µs (Figs. 7 ▸ *a* to 7 ▸ *d*), the outline of the melt pool became more evident as the visible-light photon flux collected by the camera increased by a factor of 6.8 (taking account of the APS being operated in hybrid mode), and hence the signal-to-noise ratio improved significantly. The solid–liquid and liquid–gas interfaces are demarcated symbolically in Fig. 7 ▸(*e*) by red and blue lines, respectively, for ease of visualization. Since the liquid–vapor interface in the LPBF process of Inconel was fairly stable, the increased exposure time did not introduce significant motion blur in the images. Hence, these images can be used to obtain reliable quantitative information about the melt-pool dynamics. Another avenue to increase the signal-to-noise ratio is through increasing the flux of high-energy photons by using two undulators simultaneously and adjusting the X-ray beamline components. This approach will be tried in the future to improve the image quality for heavier materials such as Ni-based superalloys and stainless steel.

Although snapshots are presented here to demonstrate the capability of the method, the entire scan length of the laser was recorded using the multi-frame high-speed camera. Hence, the evolution and dynamics of all the important physical phenomena can be tracked using the X-ray images. Further, the effects of various experimental parameters, including the laser power and scan speed, on physical phenomena, and subsequently on defects, can be investigated using the image sequences. Parametric studies of the LPBF process will be presented in future publications.

### 3.2. Ultrafast imaging of the LPBF processes   

**Figure 8:**
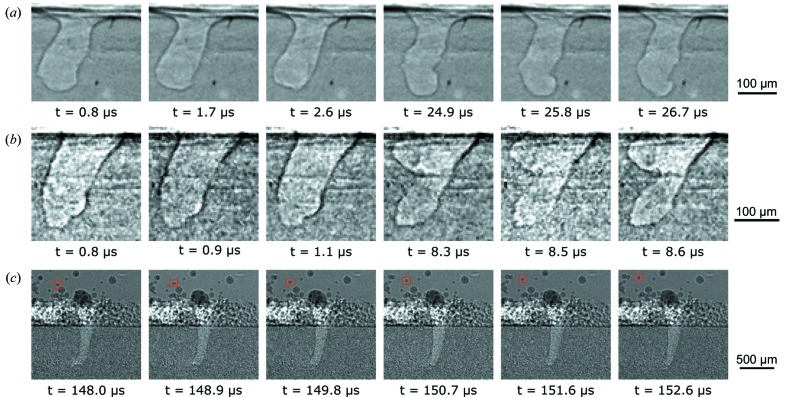
High-speed X-ray images of (*a*) and (*b*) the laser AM process for the Al–Si10–Mg plate, and (*c*) the powder-bed fusion process for Ti–6Al–4V. The frames are cropped such that the details of the vapor depression can be clearly identified as *e.g.* oscillations in the liquid–vapor surface. The experimental parameters were: (*a*) laser power 520 W, scan speed 0.8 m s^−1^, frame rate 1.08 MHz and exposure time 200 ns; (*b*) laser power 468 W, scan speed 0.6 m s^−1^, frame rate 10 MHz and exposure time 50 ns; (*c*) laser power 416 W, scan speed 0.7 m s^−1^, frame rate 1.08 MHz and exposure time 200 ns. The thickness of the plates was approximately 500 µm. The powder size for panel (*c*) was 15–45 µm.

Some ultrafast laser-melting experiments were performed with the Shimadzu HPV-X2 high-speed camera with recording rates of 1.08 million and 10 million frames s^−1^. The main aim of the ultrafast imaging experiments was to investigate some comparably dynamic phenomena in the LPBF process. Image sequences from representative experiments are presented in Fig. 8 ▸. Figs. 8 ▸(*a*) and 8 ▸(*b*) show the image sequence for the vapor depression in aluminium alloy plates recorded at 1.08 and 10 million frames s^−1^, respectively (note that 10 MHz is the camera recording speed and the X-ray pulse rate is 6.5 MHz). Fig. 8 ▸(*c*) shows an image sequence of the LPBF process in Ti–6Al–4V. Note that, in all image sequences, the time stamp displayed is with respect to the start of the recording and not to the time at which the experiment started.

In Fig. 8 ▸(*a*), two sets of three consecutive frames are presented; the complete set of ultrahigh-speed frames is presented in the video in the supporting information. The inter-frame separation for consecutive frames was 900 ns and the separation between the two sets was approximately 24 µs (27 frames). The boundary between the liquid and gas phases forming the depression can be identified clearly. The shape of the depression changed only gradually over each set of consecutive frames; however, the gradual changes accumulated over time, such that a drastic change was observed between the two sets and the large oscillations in the liquid–vapor interface are well resolved. This observation clearly shows that slower recording speeds (around 50000 frames s^−1^) can be used to probe large changes in the size and shape of the depression in aluminium alloys, yet the progression between these changes can only be recorded using sufficiently high frame rates.

For images obtained at a 10 MHz camera recording frequency (6.5 MHz X-ray pulse rate), two image sequences separated by 7.5 µs (75 frames) are presented in Fig. 8 ▸(*b*). Each image sequence presents two consecutive frames (frame separation 100 ns) and a frame separated by 200 ns from the two consecutive frames. Since the ultrahigh-speed recording was not synchronized with the X-ray pulses, approximately every third frame did not receive an X-ray pulse during the frame exposure time and the image was captured based on the afterglow effect from the previous X-ray pulse. These frames were significantly darker than the other frames since the afterglow effect was very small, as explained previously in §2.3. Although only small changes in the vapor-depression geometry were observed in the first image sequence (*t* = 0.8 to 1.1 µs), significant differences were observed in the second sequence (*t* = 8.3 to 8.6 µs). These image sequences reiterate that slower recording speeds may be sufficient for tracking large changes in the vapor depression. However, rapid geometry changes such as the oscillations in the liquid–vapor interface can only be tracked using the ultrafast recording speeds. Furthermore, some phenomena may be missed if the recording speeds are slower. It should be noted here that the 10 MHz camera recording rate is the fastest continuous recording speed that can be obtained using current commercially available CMOS-type high-speed cameras (Tochigi *et al.*, 2013 ▸; Kuroda & Sugawa, 2018 ▸).

To investigate the ultrafast dynamics of the LPBF process, a Ti–6Al–4V powder-bed system was used. The recording was performed at a frequency of 1.08 MHz. Six consecutive frames from a representative experiment are presented in Fig. 8 ▸(*c*). The vapor depression, molten metal particles on the surface and ejected powder particles can be clearly observed in the frames. One important advantage of ultrafast recording was the ability to track ejected particles with higher temporal fidelity. From preliminary calculations, the maximum velocity for the ejected particles was around 30 m s^−1^ in this particular case. The velocities reported here and previously (Zhao *et al.*, 2017 ▸) were calculated from the planar projections of the particles and only account for the motion in the projected plane. Since the velocities were calculated without taking the out-of-plane motion into account, these numbers depict the lower bound for the ejection velocities. Slower recordings (50 kHz) can still track high-velocity particles that move in a straight line. However, some phenomena such as inter-particle collisions and complex trajectories of the particles may be missed. It should be noted here that the ejection velocities are dependent on many process parameters, such as materials, laser power and scan speed.

Another way that ultrafast recording speeds can aid computerized particle tracking is by mitigating the confusion caused by rotation of particles with irregular shapes. The rotation of the particle marked by the red square in Fig. 8 ▸(*c*) is clearly evident. The angular velocity of the marked particle was approximately 3.5 × 10^5^ rad s^−1^ (90° rotation in 4.5 µs). Although these rotations can still be captured using slower recording speeds, the changing shape of the projected image of the rotating particles, along with the large separations in particle positions in slower recordings, make accurate tracking of the particles difficult. The information gleaned from the ultrafast experiments in terms of increased accuracy may then be leveraged to improve particle tracking in slower recordings.

It should be noted that the X-ray images provide geometric information (vapor-depression and melt-pool dimensions, particle-ejection trajectories) but do not provide thermal information such as temperature fields. In future, high-speed X-ray imaging could be integrated with *in situ* visible-light and thermal imaging to gain a complete understanding of the process. Further, by correlating the X-ray images with *in situ* and *operando* data obtained using other types of sensors (thermal, visible light, acoustic *etc.*), the X-ray experiments will help in developing the in-process control of the build to reduce the number of defects. From the representative results presented here, it is clear that some physical phenomena such as vapor-depression behavior and powder ejection need very high recording rates. Although rapid changes in the vapor depression can only be captured using ultrafast imaging, large-scale changes can still be captured using the lower recording speeds. Similarly, slower recordings are capable of tracking the trajectories of particles moving in a straight line, which constitutes most of the ejected particles. The information gathered from the ultrafast and slower recordings complement each other to provide a complete understanding of the LPBF processes.

## 4. Conclusions   

High-speed synchrotron X-ray imaging was used concurrently with an experimental laser setup to investigate the underlying physical phenomena in metal LPBF processes. The custom laser-melting apparatus comprised a high-power laser, a laser scanner, a vacuum-compatible experimental chamber, and alignment stages. The laser apparatus was synchronized with the high-speed X-ray imaging to capture the laser AM processes *in situ*. Two different ranges of recording speeds were used, 30000–50000 frames s^−1^ and 1.08–10 million frames s^−1^. 10 million frames s^−1^ is the fastest continuous recording rate currently available using a commercially available CMOS-type high-speed camera. This resulted in an imaging recording rate of 6.5 MHz in the current setup, which is the fastest X-ray imaging speed reported so far for studying AM processes. Many important physical phenomena involved in the process, including melt-pool dynamics and solidification, formation of porosity, vapor-depression behavior and powder ejection were recorded with high spatial and temporal resolution. Further, some of the phenomena such as vapor-depression dynamics and powder ejection exhibited fast dynamics and were identified as processes that may benefit from ultrafast recording speeds. Using ultrafast recording speeds, the rapid oscillations of the vapor depression and the high-velocity rotating powder particles were quantified for the first time.

The high-speed X-ray imaging technique will be vital for understanding the physics that governs the quality of parts manufactured using LPBF processes. Further, the results obtained using the X-ray imaging framework will also be critically important in validating the numerical models that are being developed for such processes. This experimental method will be helpful in determining the optimal processing conditions, developing new materials for AM, and investigating the new techniques for manufacturing functionally graded and multimaterial products.

## Supplementary Material

Supporting video for Figure 6(a). DOI: 10.1107/S1600577518009554/mo5183sup1.avi

## Figures and Tables

**Figure 2:**
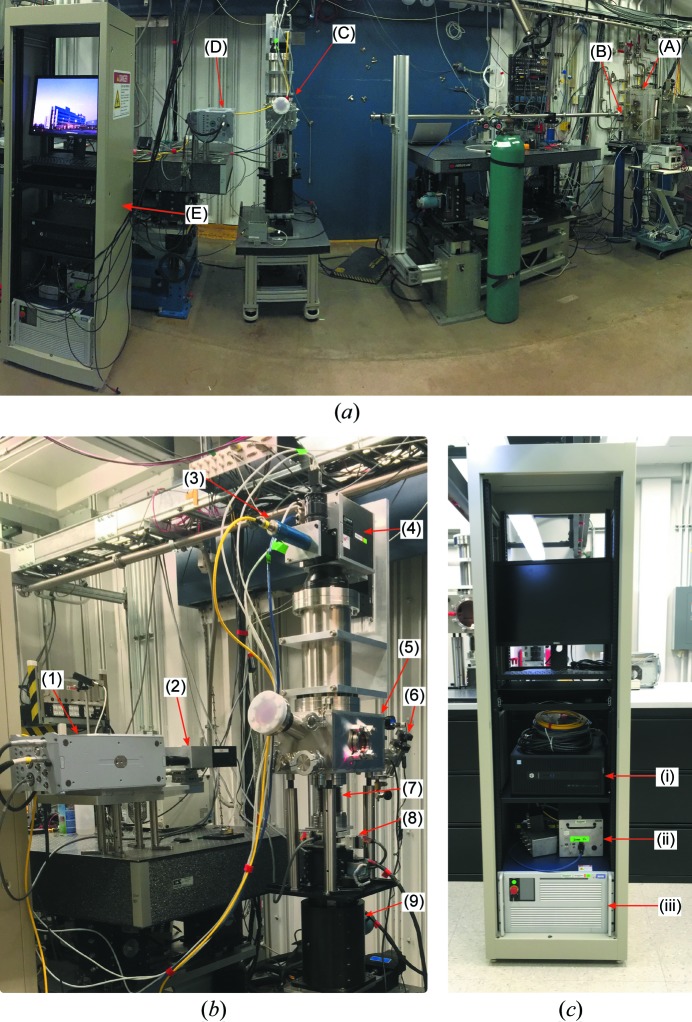
Photographs of the experimental setup. (*a*) Overview of the 32-ID-B beamline at the APS. (A) Slow shutter, (B) fast shutter, (C) laser AM experimental setup, (D) high-speed X-ray imaging setup, and (E) laser system control computer and laser source rack. (*b*) Detailed view of the setup. (1) High-speed camera, (2) scintillator–mirror–objective lens assembly box, (3) laser feeding fiber and collimator, (4) laser scanner, (5) experimental chamber, (6) connection for the vacuum pump, (7) vacuum-compatible bellows, (8) three-axis translational stages for sample manipulation, and (9) two-axis stage for laser–X-ray alignment. (*c*) A detailed view of the control rack. (i) Scanner control computer, (ii) power sources and connection boxes for the scanner, and (iii) laser source.

**Table 1:** Powders and substrates

Material	Particle size	Powder vendor	Substrate vendor
Al–10Si–Mg	15–45 µm	LPW Technology Ltd, UK	McMaster–Carr, USA
Ti–6Al–4V	15–45 µm	EOS GmbH, Germany	Titanium Distribution Services Inc., USA
Inconel 718	N/A	N/A	Manufactured using electron-beam melting process at CMU

**Table 2:** Integrated flux of the X-ray beams (1.5 mm × 1.5 mm) generated with different undulator conditions

Undulator period (cm)	Undulator gap (mm)	Integrated flux [photons s^−1^ (0.1% bandwidth)^−1^]	Single-pulse flux [photons s^−1^ (0.1% bandwidth)^−1^]
1.8	12	1.5 × 10^16^	2.3 × 10^9^
1.8	16	3.8 × 10^15^	5.8 × 10^8^
3.3	20	4.0 × 10^16^	6.1 × 10^9^
3.3	30	1.0 × 10^16^	1.5 × 10^9^

**Table 3:** Experimental settings for the high-speed cameras

Camera	Frame rate (frames s^−1^)	Frame size (pixel × pixel)	Temporal resolution (ns)	Spatial resolution (µm pixel^−1^)	APS operation mode
Photron FastCam SA-Z	3.01 × 10^4^	768 × 768	0.1 or 500	1.9	Hybrid
	4.52 × 10^4^	640 × 624	0.1 or 500	1.9	Hybrid
Shimadzu HPV-X2	1.08 × 10^6^	400 × 250	0.1	3.2	24-bunch
	1.00 × 10^7^	400 × 250	0.1	3.2	24-bunch
